# Comparison of Ventilatory and Metabolic Demands Across Percentage-Based Heart Rate Zones in Firefighters

**DOI:** 10.3390/jfmk11010102

**Published:** 2026-02-28

**Authors:** Benjamin J. Mendelson, David J. Cornell, Scott D. Brau, Nathan T. Ebersole, Robert J. Flees, Kyle T. Ebersole

**Affiliations:** 1 Human Performance & Sport Physiology Laboratory, School of Rehabilitation Sciences & Technology, University of Wisconsin-Milwaukee, Milwaukee, WI 53211, USA; 2 Health Assessment Laboratory, Department of Physical Therapy and Kinesiology, University of Massachusetts Lowell, Lowell, MA 01854, USA

**Keywords:** heart rate zones, cardiovascular workload, air consumption

## Abstract

**Background:** The purpose was to determine the ventilatory and metabolic demands in percentage-based heart rate (HR) zones in active-duty firefighters. **Methods:** Male career firefighters (*n* = 48, 38.17 ± 9.02 years, 1.79 ± 0.05 m, 88.27 ± 12.50 kg) completed a maximal treadmill test while wearing chest strap monitors to measure physiological responses corresponding to Zone 1 (50–59%), Zone 2 (60–69%), Zone 3 (70–79%), Zone 4 (80–89%), and Zone 5 (90–100%) based on age-predicted maximal HR. Aerobic capacity (VO_2PEAK_, mL·kg^−1^·min^−1^), average minute ventilation (V_E_, L·min^−1^), and respiratory exchange ratio (RER) in each zone was measured via indirect calorimetry. Linear mixed models determined significant differences in V_E_, RER, and time in zone (min). **Results:** Significant relationships emerged between VO_2PEAK_ and average RER in Zone 5 (*r* = −0.33) and time in Zone 3 (*r* = 0.45), Zone 4 (*r* = 0.41), and Zone 5 (*r* = 0.41). A significant HR zone effect emerged in V_E_ (*F* = 516.01, *p* < 0.001) indicating that V_E_ increased as zone intensity increased. After controlling for VO_2PEAK_, a significant HR zone effect emerged in RER (*F* = 11.90, *p* < 0.001), indicating that average RER increased as zone intensity increased. No HR zone effect was found for time in zone (*F* = 1.18 *p* = 0.332) after controlling for VO_2PEAK_. **Conclusions:** A practical cardiovascular workload measure, such as percentage-based HR zones determined from treadmill testing, have distinct ventilatory and metabolic responses. Higher aerobic capacity is related to greater time spent working in higher HR zones.

## 1. Introduction

Firefighting is strenuous and dangerous work that can predispose first responders to high risk of occupational line-of-duty injuries. As recently as 2023, it was reported that overexertion and strain-related injuries were the leading causes of non-fatal (31% of total) and fatal (54% of total) line-of-duty injuries [1,2]. Due to these injuries occurring within the line of duty, conceptualization of firefighting has advanced in the recent literature to better understand the workload experienced by firefighters. Workload measurement is divided into external load, or the quantifiable amount of work completed, and internal load, or the psychophysiological response to the external load [3,4]. In the sport-athlete literature, external load may be measured by load lifted in resistance training, or the distance covered during a training session or competition via wearable global positioning system tracking devices [4,5]. Internal load is then representative of the body’s response to this load, which can be represented by heart rate (HR), respiration rate, oxygen consumption (VO_2_), or perceived exertion [3]. Workload monitoring in the fire service has incorporated various methods of tracking external and internal loads.

External load is often represented by the time elapsed while performing job tasks, which has also been used to quantify firefighter performance [6,7,8,9,10,11]. While time to complete simulated tasks is often used to assess levels of ability or performance in firefighters, time spent working has a unique role in the total work demands of this population. It has been previously noted that firefighters responding to structure fire emergencies may have work durations that range between 8 and 28 min in singular work bouts during an emergency call response [12]. However, it should be noted that firefighters may perform repeated work bouts while on-scene, as it has been reported that the average length of medical and fire calls in career firefighters at a career metropolitan department in the United States ranged between 14.30 and 42.75 min [6]. In addition, firefighters are required to wear full personal protective equipment (PPE) ensembles, which includes a helmet, turnout coat, pants, boots, gloves, hood, and a specialized self-contained breathing apparatus (SCBA) and respirator, which can add between 20 and 34 kg on top of body mass [13,14,15]. The impact of this added load can be an impairment of movement ability, balance, and an increase in the internal load response to work including an increased rate of oxygen consumption [13,14,16,17].

The increased rate of oxygen consumption directly impacts on the workability of a firefighter. In accordance with the National Fire Protection Association (NFPA) 1970 Standard, the SCBA units worn by members must be rated by the National Institute for Occupation Safety and Health (NIOSH) to last a minimum of 30 min, with configurations commonly allowing 30, 45, or 60 min of air [18,19]. The NIOSH time-based rating is based on a fixed ventilation rate of 40 L·min^−1^, yet prior studies examining firefighter respiratory demands utilizing portable metabolic analysis have reported ventilation rates ranging on average of 53.3–96.2 L·min^−1^ during simulated task performance [19,20,21,22,23,24,25]. It should be noted that these prior simulated task studies were not completed using live fire scenarios, yet fire calls have been indicated to demand near-maximal internal load, as measured by HR or time spent in HR zones greater than 80% of maximal [6,12,26,27]. As such, there is a need to explore practical methods to enhance firefighter internal load monitoring to incorporate ventilation rate.

Internal load tracking in firefighters often uses HR monitoring to identify the range of cardiovascular intensities experienced by members. The incorporation of training impulse (TRIMP)-based algorithms have grown in recent studies to examine the impact of external (e.g., time) and internal (e.g., HR) load in firefighters within a singular algorithm [7,28,29]. One such algorithm is the Edward’s Training Impulse (eTRIMP) which accounts for time spent in five percentage-based zones from 50 to 100% in 10% increments determined from an individual’s age-predicted or achieved maximal HR [30]. This algorithm has been used to calculate cardiovascular workload in firefighters in simulated tasks and on-duty emergency calls, and is a practical measure, as it does not require the need for laboratory testing of ventilatory thresholds such as with the Lucia’s TRIMP [6,7,29,31]. However, it has been suggested that eTRIMP may lack specificity of physiological intensity, in that there is a lack of understanding how percentage-based zones align with ventilatory or metabolic physiological responses [3].

Thus, the purpose of this study was to explore the ventilatory and metabolic responses across percentage-based zones in active-duty firefighters in an incremental treadmill task. A laboratory maximal treadmill test was used to identify significant differences in ventilatory and metabolic demands occurring across zones via minute ventilation (V_E_, L·min^−1^) and respiratory exchange ratio (RER), respectively. In addition, a secondary purpose of this study was to examine the relationship of peak aerobic capacity (VO_2PEAK_, mL·kg^−1^·min^−1^) on the ventilatory and metabolic responses in zones, as VO_2PEAK_ is a fitness factor directly outlined in the NFPA 1580 Standard that firefighters should optimize for job readiness [32]. To date, VO_2PEAK_ has demonstrated moderate negative relationships to time to complete simulated tasks and moderate relationships with SCBA depletion [22,24,33,34], suggesting that higher fitness is related to faster performance and better air management, yet it is unknown whether VO_2PEAK_ is related to relative ventilatory and metabolic intensity.

## 2. Materials and Methods

### 2.1. Experimental Approach

Active-duty firefighters were recruited from career fire departments in a metropolitan area in the midwestern United States. All participants completed a single data-collection session including anthropometric measures of height (m), body mass (kg), and body mass index (BMI, kg·m^−2^) to determine body composition. An incremental maximal treadmill test (TM) using direct capture of expired air through a calibrated metabolic cart system was conducted to determine VO_2PEAK_ (mL·kg^−1^·min^−1^) and maximal heart rate (HR_PEAK_, bpm) using chest-strap heart-rate monitors. Data from the metabolic cart was used post hoc to calculate percentage-based heart-rate zones based on age-predicted maximal heart rate (MHR), and the corresponding average V_E_ (L·min^−1^), RER, and time (min) spent in each zone were extracted as well. Approval for this study was granted by the Institutional Review Board at the University of Wisconsin-Milwaukee (Protocol Numbers 17.243, 23.058). Participants were instructed to abstain from excess caffeine intake and strenuous exercise prior to the data collection session.

### 2.2. Subjects

A sample of male career active-duty firefighters (*n* = 48) volunteered to participate in this study. Prior to data collection, participants were screened for eligibility. To be considered eligible for this study, participants were required to be non-probationary active-duty firefighters at the rank of firefighter, heavy equipment operator, lieutenant, or captain over the age of 18 years old. Participants must not have had an injury in the prior three months and not be diagnosed with any cardiovascular, metabolic, or neuromuscular conditions. In addition, participants were required to be completing steady-state running (e.g., at least 30 minutes at a frequency of 1–2 sessions per week) as a component of their normal exercise regimen.

### 2.3. Procedures

#### 2.3.1. Maximal Treadmill Test (TM)

The incremental maximal treadmill test followed the protocol designed by the International Association of Fire Fighters and International Association of Fire Chiefs Joint Labor-Management Wellness Fitness Initiative (WFI) [35]. Participants completed this protocol on a motorized treadmill (Woodway 4Front, Woodway USA Inc., Waukesha, WI, USA), beginning with a three-minute walk at 3.0 mph at a 0% grade, followed by an increase in speed to 4.5 mph for one minute. The test then progressed in one-minute stages where treadmill incline was increased by 2% or treadmill velocity was increased by 0.5 mph at alternating time points [36,37]. While the WFI protocol is designed to be submaximal and terminate when participants achieve 85% of age-predicted maximal heart rate (MHR = 208 − 0.7 × age, bpm) to estimate VO_2PEAK_, participants of this study continued on until they achieved at least two of three criteria for termination: (a) meeting or exceeding predicted MHR for more than 15 seconds, (b) participant rating of perceived exertion was ≥10 on the Borg CR-10 RPE scale [38], and (c) participant terminated the test voluntarily due to fatigue [36,37,39,40].

Before the TM, each participant was fitted with a mouthpiece and headgear that included a two-way non-rebreathing valve to ensure that all expired air during the test was directed into a calibrated metabolic cart system (PARVO TrueOne 2400, ParvoMedics, Inc., Sandy, UT, USA). In addition to the direct breath-by-breath capture, participants wore chest-strap heart-rate (HR) monitors (Polar H10, Polar Electro Oy, Kempele, Finland). Data were expressed as 15 s averages across the duration of the test, and the highest 15 s average VO_2_ value represented VO_2PEAK_ and the highest 15 s average heart-rate value represented peak HR (HR_PEAK_, bpm).

#### 2.3.2. Determination of Physiologic Responses in Heart-Rate Zones

Percentage-based zones were determined by using age-predicted MHR. Zones were identified as Zone 1 (50–59% MHR), Zone 2 (60–69% MHR), Zone 3 (70–79% MHR), Zone 4 (80–89% MHR), and Zone 5 (90–100% MHR) [30]. Average V_E_ (L·min^−1^), RER, and time spent working (min) in each zone were determined post hoc using data from the metabolic cart system, providing ventilatory and metabolic context to each zone. The average ventilatory, metabolic, and time-based response within each zone was then included in data analysis.

### 2.4. Statistical Analysis

Prior to analysis, all data were inspected and confirmed for normality via visual analysis of Q-Q plots. A paired *t*-test was used to examine significant differences between estimated MHR and HR_PEAK_ achieved on the TM. Pearson correlations were used to examine for significant relationships between VO_2PEAK_ and the average V_E_, RER, and time in Zones 1–5 during the TM. Separate linear mixed models were conducted using restricted maximum likelihood estimation to examine for changes in the ventilatory, metabolic, or time-based response across HR zones. If significant relationships were found between VO_2PEAK_ and average V_E_, RER, or time in zones, VO_2PEAK_ was added as a fixed effect to account for the impact of aerobic capacity. Correlation coefficients were assessed as negligible (*r* = 0.00–0.10), weak (*r* = 0.10–0.39), moderate (*r* = 0.40–0.69), strong (*r* = 0.70–0.89), or very strong (*r* = 0.90–1.00) [41]. The alpha level was set a priori to α = 0.05 for the linear mixed models. Post hoc pairwise comparisons of VE, RER, and time in HR zones in significant linear mixed models were conducted using Bonferroni tests. Analysis and data visualizations were conducted using R Statistical Software (version 4.4.1) [42] and IBM Statistical Package for the Social Sciences, version 29.0.1.0 (IBM SPSS Statistics for Windows, Version 29.0, IBM Corp: Armonk, NY, USA).

## 3. Results

Descriptive statistics for anthropometric and TM results are presented in Table 1 for the study participants. Based on age-based normative values for VO_2PEAK_ from the American College of Sports Medicine (ACSM), participants ranged from Very Poor (*n* = 3), Poor (*n* = 4), Fair (*n* = 12), Good (*n* = 19), Excellent (*n* = 9), and Superior (*n* = 1) [43]. Based on ACSM norms, participants in the Poor and Very Poor categories fall below the age-based 35th percentile, meaning that seven participants would fall below the aerobic capacity recommendation in the NFPA 1580 standard [32]. in regard to BMI, participants ranged from Normal (*n* = 10, 18.5 < BMI < 24.9), Overweight (*n* = 31, 25.0 < BMI < 29.9), Obese Class I (*n* = 4, 30.0 < BMI < 34.9), Obese Class II (*n* = 1, 35.0 < BMI < 39.9), and Obese Class III (*n* = 2, BMI ≥ 40.0) [43]. Results of the paired *t*-test (*t*_47_ = −1.796, *p* = 0.079) indicated that HR_PEAK_ was not significantly different from predicted MHR (183.6 ± 10.2 vs. 181.37 ± 6.32, respectively).

**Figure 1:**
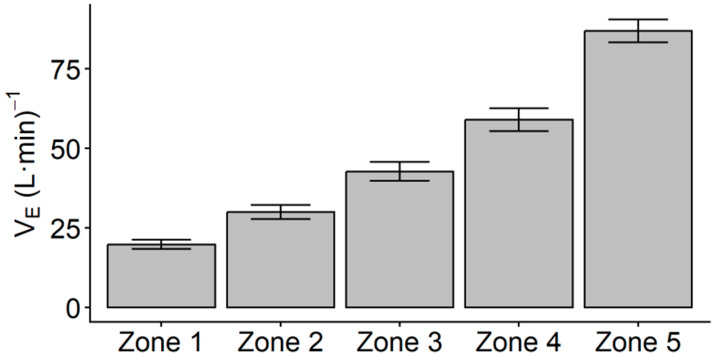
Plots of linear mixed model estimated marginal means ± 95% CI for minute ventilation (V_E_) after controlling for VO_2PEAK_. Post hoc Bonferroni pairwise comparisons indicated significant differences in the average V_E_ across all HR zones (*p* < 0.001).

Unadjusted descriptive statistics for V_E_, RER, and time in zones are presented in Table 2. Pearson product correlations indicated no significant relationship between VO_2PEAK_ and V_E_ in any HR Zone. Results of the linear mixed model indicated a main effect of HR zone on V_E_ (*F*_4,47_ = 516.01, *p* < 0.001). Post hoc pairwise comparisons indicated that all estimated marginal mean V_E_ values for each HR zone were significantly different from each other (*p* < 0.001), with V_E_ significantly increasing across each HR zone (Figure 1).

**Figure 2:**
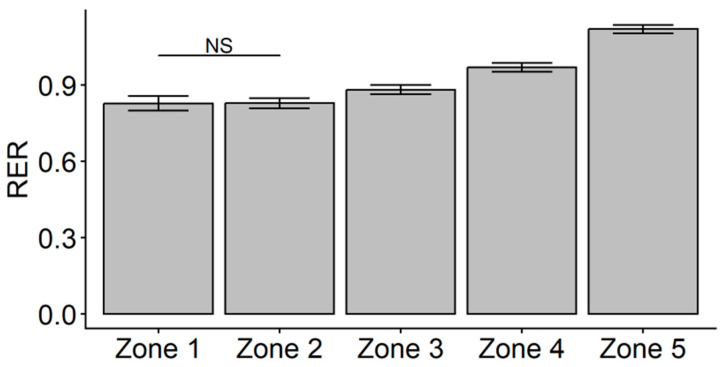
Plot of linear mixed model estimated marginal means ± 95% CI for respiratory exchange ratio (RER) after controlling for VO_2PEAK_. Post hoc Bonferroni pairwise comparisons indicated no significant difference (NS) between the average RER in Zone 1 and Zone 2, but all other comparisons were significant (*p* < 0.001).

Pearson correlations indicated that VO_2PEAK_ was weakly related to RER in HR Zone 5 (*r* = −0.328, *p* = 0.023) but was not related to RER in any other HR Zone. A linear mixed model with fixed effects for HR zone and VO_2PEAK_ indicated that, after controlling for the effect of VO_2PEAK_ (*F*_1,46_ = 2.007, *p* = 0.163), there was a main effect of zone on RER (*F*_4,46_ = 11.904, *p* < 0.001). Follow-up pairwise comparisons indicated that the estimated marginal mean value of RER in Zone 1 was not significantly different from Zone 2, but all other HR zone comparisons were different (*p* < 0.001). After controlling for VO_2PEAK_, the average RER in each HR Zone was not different between Zone 1 and Zone 2 but significantly increased from Zone 2 to Zone 5 (Figure 2). Further, higher cardiorespiratory fitness was related to a lower average in RER in Zone 5.

**Figure 3:**
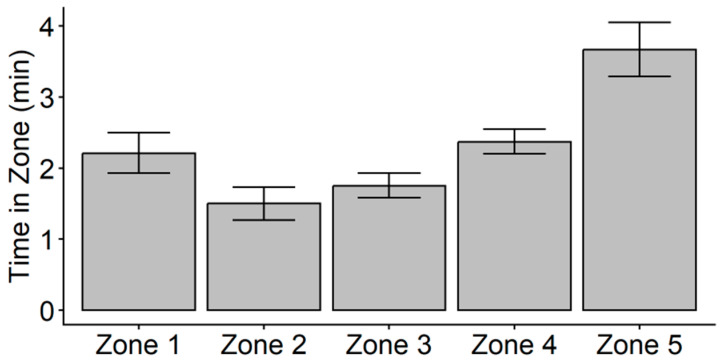
Plot of linear mixed model estimated marginal means ± 95% CI for time in zone after controlling for VO_2PEAK_. After controlling for VO_2PEAK_, there was no significant main effect of time (*p* = 0.332).

It was found that VO_2PEAK_ was moderately related to mean time spent in Zone 3 (*r* = 0.448, *p* = 0.001), Zone 4 (*r* = 0.413, *p* = 0.004), and Zone 5 (*r* = 0.410, *p* = 0.004). Due to these significant relationships, a follow-up linear mixed model was conducted to examine significant differences in time spent in each of the five HR zones with VO_2PEAK_ added as a fixed effect. The results indicated that, after controlling for the influence of VO_2PEAK_ on time (*F*_1,46_ = 39.372, *p* < 0.001), there was no longer a significant main effect of HR zone on time (*F*_4,46_ = 1.181, *p* = 0.332). These results suggest that higher cardiorespiratory fitness was associated with greater time spent in Zones 3–5 on the TM. Yet, when cardiorespiratory fitness was controlled for, there was no significant difference in the average time spent in any of the five HR zones during the TM (Figure 3).

## 4. Discussion

The purpose of this study was to examine the ventilatory, metabolic, and time-based performance across percentage-based zones of heart rate (HR) intensity in active-duty firefighters during a maximal treadmill test. Measurement of time spent in HR intensity zones has been used in the fire service to conceptualize the cardiorespiratory demand of simulated and on-site emergency response [6,7,24,28,29,44]. A percentage-based zone approach provides practitioners with a non-invasive method to determine HR zones, in contrast to other methods of quantifying cardiovascular intensity zones that require testing to determine ventilatory thresholds or use of heart rate reserve (HRR) [3,30,31]. While it has been suggested that there is limited evidence supporting links between percentage-based ranges and metabolic intensity, the results of the current study suggest that percentage-based HR zones ranging from 50 to 100% have distinct ventilatory (i.e., V_E_) and metabolic (i.e., RER) demands when determined from an incremental maximal treadmill task [3]. Thus, the use of percentage-based zones may be useful for understanding the ventilatory and metabolic demands of firefighters when performing work.

It was reported by Marciniak et al. [6] that, in active-duty structural firefighters responding to medical (MED) emergency calls or fire calls that did not require fire suppression and/or auto extrication (FIRE0), the cardiovascular intensity was predominately in Zone 1 (21.89 ± 15.65% of average time per MED, 27.55 ± 19.23% of average time per FIRE0). It was also noted that a very small average percentage of call duration was in Zone 4 for MED (0.14 ± 0.24%) or FIRE0 (0.56 ± 1.12%) and there was no time spent in Zone 5 for MED or FIRE0. However, for fire calls that did require fire suppression and/or auto extrication (FIRE1), while the highest average percent of call duration was in Zone 2 (20.07 ± 17.10%), approximately 16% of the average percentage of call duration was between Zone 4 and Zone 5. It should be noted that the investigation by Marciniak et al. [6] quantified total workload for an entire call, which may include intermittent bouts of work and rehabilitation. According to the NFPA 1580 Standard, firefighters must take a minimum 20 min rehabilitation following the use of an SCBA tank or performing 40 minutes of strenuous work without using an SCBA [32].

The respiratory demands of firefighting are of substantial practical interest, as a key component of firefighter PPE includes the SCBA. The SCBA units provide a finite amount of compressed air for firefighters to breathe to avoid exposure to hazardous environments. Per the NFPA 1970 Standard, firefighters must use SCBA tanks that are rated by the National Institute of Occupational Safety and Health (NIOSH) to last for a minimum of 30 minutes [18]. This time-based rating is made using a standard respiratory flow rate of 40 L·min^−1^ of air consumed, and a practical example noted by Norris et al. [25] was that a 4500 psi SCBA tank contains approximately 1869 L of compressed air, and would last about 45 minutes at a flow rate of 40 L·min^−1^ [45]. In the context of the results of this study, this 40 L·min^−1^ would align with approximately a Zone 2 or Zone 3 intensity on a laboratory-controlled graded maximal treadmill test.

Prior studies have demonstrated that firefighters exceed this standard flow rate and reach near-maximal heart rate intensities when performing simulated fireground work, both in non-fire and live-fire scenarios [12,22,24,27,29,44]. In a study that utilized portable gas analyzers to track V_E_ in active-duty firefighters (*n* = 33) performing a simulated high-rise scenario, Williams-Bell et al. [23] reported that average V_E_ ranged from 85.3 to 90.7 L·min^−1^, more than double the standard flow rate, and average HR ranged from 80 to 96% of maximal HR across the scenario, or within Zones 4–5 using a percentage-based zone model. Similar findings were reported by Langford et al. [20] in a study that also used portable gas analyzers to assess air consumption in active-duty firefighters (*n* = 44) performing an obstacle-course format simulated air consumption drill, where the average V_E_ was reported as 96.2 ± 13.0 L·min^−1^ and average HR was 91.4 ± 6.0% of maximal HR, corresponding with Zone 5. A separate investigation by Langford et al. [21] in active-duty firefighters (*n* = 40) performing the obstacle-course format simulated air-consumption drill at a standardized pace also demonstrated an average V_E_ of 87.4 ± 11.4 L·min^−1^ and average HR of 87.5 ± 7.3% of maximal HR, or within Zone 4. Interestingly, these findings correspond with the V_E_ associated with HR Zone 5 from the current investigation but the HR response aligns with HR intensities between Zone 4–5. An investigation by Jagim et al. [29] examining active-duty firefighters (*n* = 57) performing a simulated air-management course that lasted an average of about 25.17 ± 4.42 min reported that participants spent the largest percent of time on the course in Zone 4 (35.2 ± 15.9% of total time) and Zone 5 (45.1 ± 25.7% of total time). Additionally, it was reported by Williams-Bell et al. [22] that active-duty firefighters (*n* = 33, 40.7 ± 6.5 years) performing a simulated search-and-rescue task in a subway system experienced an average of approximately 76 ± 7% of maximal HR while performing seven different sequential tasks, or within Zone 3 intensity. Two of the tasks, a guided rescue and one-story stair ascent, elicited an average of 83 ± 7% and 81 ± 6% of maximal HR, respectively. In addition, it was noted in this study that metabolic intensity of the entire subway system task produced an average RER of 0.95 ± 0.08. These results correspond with the findings of the current study, in that intensity in HR Zones 3–4 on the treadmill test elicited a metabolic intensity that favors anaerobic energy systems or predominantly carbohydrate metabolism.

The overlap present between the V_E_ and HR that has been previously reported in simulated fireground tasks and in the current investigation during a laboratory-controlled maximal treadmill task suggests that the use of percentage-based HR zones may provide meaningful insight to during-task ventilatory demand. However, the discrepancies seen between prior findings and the current study may be potentially due to added neuromuscular load or thermal strain of PPE, which has been noted to increase relative demand of firefighting work as added external load and impact regulation of body heat [13]. Further, the results of the current study suggest that HR Zone 3 on a maximal treadmill test may have a lower average RER when compared to the overall average RER reported by Williams-Bell et al. [22]. This may also be attributed to differences in motor unit recruitment and activation of Type II muscle fibers that prefer anaerobic metabolic systems to support the increase in neuromuscular demand from the added load of PPE [13,14,16,46].

While factors such as PPE or environmental conditions may impact the neuromuscular or cardiorespiratory response to fireground tasks, a consistent research finding has been that improved cardiorespiratory fitness is beneficial to performance and recovery. Performance in firefighter-specific contexts has often been conceptualized as a quicker time to complete simulated tasks, and higher aerobic capacity has been consistently linked to faster time to complete tasks [9,33,34,47,48]. In the recent literature exploring the impact of aerobic capacity on air consumption, it was reported that higher aerobic capacity was moderately related to greater Work Efficiency and lower overall cardiorespiratory strain as represented by time-strain-air (TSA) score in simulated non-fire and live-fire environments [24,25,44]. It should be noted that both the Work Efficiency measure introduced by Norris et al. [25] and the TSA score introduced by Windisch et al. [24] utilize total depletion of the SCBA as a measure of air consumption, as opposed to minute ventilation as measured in this study. While the results of the current investigation suggest that maximal aerobic capacity may not be related to during-task ventilation rate, it was reported by Windisch et al. [44] that maximal aerobic capacity did have a moderate relationship to lower relative heart rate during non-fire (*r* = −0.593, *p* < 0.05) and live-fire (*r* = −0.693, *p* < 0.01) simulated tasks. When considering the results of the current investigation that lower HR zones (e.g., Zones 1–2) have a significantly lower V_E_ response than higher HR zones (e.g., Zones 4–5; Figure 1), it is possible that the relationship between higher maximal aerobic capacity and lower during-task relative cardiovascular intensity may indirectly lead to lower during-task ventilatory demand. It is also possible that the links between cardiorespiratory fitness and quicker time to complete tasks may account for some of the relationship between greater aerobic capacity and lower total air depletion. However, additional work is needed to establish this relationship in fireground tasks, as other fitness factors such as neuromuscular strength, endurance, and power have also demonstrated significant relationships to faster task completion time [8,10,11,49,50].

In addition to task performance, higher aerobic capacity has also been demonstrated to be impactful to post-task autonomic nervous system (ANS) recovery. It was reported by Cornell et al. [39] that firefighters with higher cardiorespiratory fitness (VO_2PEAK_ > 42.0 mL·kg^−1^·min^−1^) displayed a significantly faster heart rate recovery (HRR) decay rate after an incremental maximal treadmill task when compared to less fit firefighters, after controlling for age and BMI. This finding was not significant after a submaximal step test, indicating that optimizing modifiable factors of body composition and cardiorespiratory fitness may impact the sympathetic nervous system (SNS) withdrawal after higher intensity tasks. ANS recovery has been reported to differ between submaximal and maximal tasks as well as incremental maximal and all-out tasks in firefighters, such that maximal tasks may require a longer time to reach baseline, and tasks with a rapid onset of severe-intensity activity may inhibit heart-rate recovery to baseline within 5–10 min [51,52]. However, when considering the prior results indicated by Windisch et al. [44], it is possible that an influence of aerobic capacity may be to lower the relative demand of tasks, which may then improve recovery during post-task rehabilitation. This requires further exploration of recovery after fireground tasks yet may have important applications to the role of improved cardiorespiratory capacity and on-site rehabilitation for firefighters.

While these findings present meaningful outcomes for understanding ventilatory and metabolic responses in percentage-based HR zones, there are limitations that require further consideration. First, the sample used in this investigation was entirely male career firefighters. While career fire departments in the United States have been reported to be comprised by approximately 95% males, the lack of female representation in this sample presents a limitation to generalizing these results to all career firefighters [53]. As such, future research should seek to replicate these methods in female firefighters. In addition, due to the conditions of the laboratory-controlled treadmill test, these results may not be fully reflective of the complex demands of fireground work. Yet, when considering the laboratory-controlled flow rate of 40 L·min^−1^ used by NIOSH, these results from a laboratory-controlled treadmill test present a need for future research to investigate the ventilatory and metabolic demands of firefighters when performing fireground work including the relevant environmental stressors, such as PPE, complex tasks, or heat stress from live-fire scenarios.

## 5. Conclusions

The results of the current investigation, when considered with prior work, suggest that the 40 L·min^−1^ standard flow rate used to determine time-based ratings of SCBA tanks aligns with a Zone 2–3 intensity. However, firefighters can routinely experience intensities of Zone 4 and Zone 5 when performing work in simulated and actual emergencies, corresponding to flow rates more than doubling the standard flow rate. This would practically lead to a faster time to deplete an air tank during intense exertion. These results suggest that utilizing a percentage-based zone delineation is a meaningful method to conceptualize cardiovascular intensity in firefighters, and modifiable factors of cardiorespiratory fitness may need to be considered as key elements of fitness training to improve overall physical work capacity.

## Figures and Tables

**Table 1:** Descriptive statistics of anthropometric and TM results.

	Mean ± SD	Range
Age (years)	38.17 ± 9.02	22.00–55.00
Height (m)	1.79 ± 0.05	1.65–1.87
Body mass (kg)	88.27 ± 12.50	65.50–130.18
BMI (kg·m^−2^)	27.87 ± 4.21	20.67–42.60
Time of test (min)	12.47 ± 1.94	7.48–16.01
VO_2PEAK_ (mL·kg^−1^·min^−1^)	44.97 ± 7.22	27.10–58.10
HR_PEAK_ (bpm)	183.60 ± 10.21	159.00–202.00

**Table 2:** Unadjusted descriptive statistics (Mean ± SD) of V_E_, RER, and time in each HR zone during the TM.

	V_E_ (L·min^−1^)	RER	Time in Zone (min)
Zone 1 (50–59% MHR)	19.77 ± 5.00	0.83 ± 0.10	2.21 ± 0.98
Zone 2 (60–69% MHR)	29.97 ± 7.66	0.83 ± 0.07	1.50 ± 0.78
Zone 3 (70–79% MHR)	42.71 ± 10.26	0.88 ± 0.07	1.75 ± 0.67
Zone 4 (80–89% MHR)	58.98 ± 12.32	0.97 ± 0.06	2.37 ± 0.65
Zone 5 (90–100% MHR)	86.89 ± 12.32	1.12 ± 0.06	3.67 ± 1.43

## Data Availability

The data presented in this study are available on request from the corresponding authors. The data are not publicly available due to privacy reasons.

## Abbreviations

The following abbreviations are used in this manuscript:

ACSM	American College of Sports Medicine
BMI	Body Mass Index
eTRIMP	Edward’s Training Impulse
FIRE0	Emergency fire call without fire suppression or automobile extrication
FIRE1	Emergency fire call with fire suppression or automobile extrication
HR	Heart rate
HR_PEAK_	Peak heart rate
MED	Emergency medical call
MHR	Age-predicted maximal heart rate
NFPA	National Fire Protection Association
NIOSH	National Institute for Occupational Safety and Health
PPE	Personal protective equipment
RER	Respiratory exchange ratio
SCBA	Self-contained breathing apparatus
TRIMP	Training Impulse
TSA	Time-Strain-Air
V_E_	Minute ventilation
VO_2_	Volume of oxygen consumed
VO_2PEAK_	Maximal aerobic capacity
WFI	Wellness Fitness Initiative
